# Cholesterol levels and long-term rates of community-acquired sepsis

**DOI:** 10.1186/s13054-016-1579-8

**Published:** 2016-12-23

**Authors:** Faheem W. Guirgis, John P. Donnelly, Sunita Dodani, George Howard, Monika M. Safford, Emily B. Levitan, Henry E. Wang

**Affiliations:** 1 Department of Emergency Medicine, University of Florida College of Medicine, Jacksonville, FL USA; 2 Department of Emergency Medicine, University of Alabama School of Medicine, Birmingham, AL USA; 3 Department of Epidemiology, University of Alabama at Birmingham, Birmingham, AL USA; 4 Department of Medicine, University of Alabama School of Medicine, Birmingham, AL USA; 5 Department of Medicine, Weill Cornell Medical College, New York, NY USA; 6 Department of Epidemiology, University of Florida, Gainesville, FL USA; 7 Department of Family Medicine, Mayo Clinic, Jacksonville, FL USA; 8 Department of Biostatistics, University of Alabama at Birmingham, Birmingham, AL USA; 9 Department of Emergency Medicine, University of Alabama at Birmingham, 619 19th Street South, OHB 251, Birmingham, AL 35249 USA

**Keywords:** Lipids, Prevention, Infection, Inflammation

## Abstract

**Background:**

Dyslipidemia is a risk factor for cardiovascular disease, with elevated low-density lipoprotein cholesterol (LDL-C) and decreased high-density lipoprotein cholesterol (HDL-C) recognized as risk factors for acute coronary events. Studies suggest an association between low cholesterol levels and poor outcomes in acute sepsis. We sought to determine the relationship between baseline cholesterol levels and long-term rates of sepsis.

**Methods:**

We used data from the Reasons for Geographic and Racial Differences in Stroke (REGARDS) cohort, a population-based cohort of 30,239 community-dwelling adults. The primary outcome was first sepsis event, defined as hospitalization for an infection with the presence of ≥2 systemic inflammatory response syndrome criteria (abnormal temperature, heart rate, respiratory rate, white blood cell count) during the first 28 hours of hospitalization. Cox models assessed the association between quartiles of HDL-C or LDL-C and first sepsis event, adjusted for participant demographics, health behaviors, chronic medical conditions, and biomarkers.

**Results:**

We included 29,690 subjects with available baseline HDL-C and LDL-C. There were 3423 hospitalizations for serious infections, with 1845 total sepsis events among 1526 individuals. Serum HDL-C quartile was not associated with long-term rates of sepsis (hazard ratio (HR) (95% CI): Q1 (HDL-C 5–40 mg/dl), 1.08 (0.91–1.28); Q2 (HDL-C 41–49 mg/dl), 1.06 (0.90–1.26); Q3 (HDL-C 50–61 mg/dl), 1.04 (0.89–1.23); Q4, reference). However, compared with the highest quartile of LDL-C, low LDL-C was associated with higher rates of sepsis (Q1 (LDL-C 3–89 mg/dl), 1.30 (1.10–1.52); Q2 (LDL-C 90–111 mg/dl), 1.24 (1.06–1.47); Q3 (LDL-C 112–135 mg/dl), 1.07 (0.91–1.26); Q4, reference).

**Conclusion:**

Low LDL-C was associated with higher long-terms rates of community-acquired sepsis. HDL-C level was not associated with long-term sepsis rates.

**Electronic supplementary material:**

The online version of this article (doi:10.1186/s13054-016-1579-8) contains supplementary material, which is available to authorized users.

## Background

One of the greatest accomplishments for preventive medicine has been the recognition and treatment of hyperlipidemia to prevent coronary artery disease (CAD). At the forefront has been the institution of HMG-CoA reductase inhibitor (statin) drug therapy to lower low-density lipoprotein cholesterol (LDL-C) to target levels with significant benefit [1]. More controversial, however, has been the association between lower levels of high-density lipoprotein cholesterol (HDL-C) and increased risk of CAD. Atherosclerosis occurs when LDL-C particles become trapped in the subendothelial space of human artery cell walls and attract inflammatory cells. Generally, HDL-C is protective because it transfers LDL-C to the liver and adrenal glands and facilitates its elimination in the intestinal tract. HDL-C also has several antioxidant and anti-inflammatory properties which can help prevent LDL-C oxidation and inflammatory cell migration. The landmark Framingham study demonstrated that low HDL-C was a risk factor for CAD death in both males and females [2]. However, recent studies found no additional benefit to raising serum HDL-C levels with medications in patients with optimized LDL-C already on statins [3].

In addition to CAD, manipulation of cholesterol levels may also play an important role in combating sepsis, the syndrome of dysregulated response to microbial infection. Sepsis results in approximately 570,000 annual emergency department (ED) visits in the United States [4]. Nearly 215,000 patients die from sepsis each year [5] and sepsis has an estimated cost of $16.7 billion annually in the United States alone [6]. The most compelling evidence for the role of lipids in protection against sepsis involves HDL-C. There are several mechanisms by which HDL-C has been shown to be protective in sepsis, including bacterial toxin binding and disposal [7, 8], monocyte suppression, macrophage and dendritic cell migration, release of inflammatory cytokines [9, 10], and inhibition of vascular and intercellular adhesion molecule expression [11]. All of these processes can lead to dysregulated inflammation and endothelial injury, which can result in the clinical manifestations of organ damage and death. Low HDL-C levels have also been associated with poor sepsis outcomes [12–14]. In addition, LDL-C has been shown to facilitate bacterial toxin clearance in sepsis [15]. Lagrost et al. [16] demonstrated that lower baseline total cholesterol levels were present in patients who developed sepsis after cardiac surgery.

In prior studies, we have demonstrated that cardiovascular disease is a risk factor for incident sepsis events [17]. The association between baseline cholesterol and rates of sepsis remains unknown. As with CAD, a relationship between cholesterol and sepsis could provide an opportunity for potentially reducing an individual’s long-term risk through targeting of particular lipids. In this study, we sought to determine the association between baseline lipid levels and long-term rates of sepsis among community-dwelling adults in the Reasons for Geographic and Racial Disparities in Stroke (REGARDS) cohort.

## Methods

### Study design

We performed a prospective cohort study using data from the REGARDS study, a national population-based longitudinal cohort.

### Data source

REGARDS is a 30,239-participant cohort study of community-dwelling adults ≥ 45 years of age. The cohort recruited participants from 2003 to 2007 [18]. The cohort is 45% male, 41% African American, and 69% aged >60 years. The study oversampled Black individuals and those living in the southeastern United States, with 21% of the cohort originating from the coastal plains of North Carolina, South Carolina, and Georgia (the “stroke buckle”), and 35% from the remainder of North Carolina, South Carolina, and Georgia plus Tennessee, Mississippi, Alabama, Louisiana, and Arkansas (the “stroke belt”).

Baseline data obtained upon participant enrollment included sociodemographics, health behaviors, chronic medical conditions, as well as information on diet, family history, psychosocial factors, and prior residences. In addition, blood and urine specimens were obtained from each participant. The study contacts participants every 6 months to determine the date, location, and reason for all hospitalizations.

### Identification of sepsis events

We identified all hospitalizations attributed by participants to a serious infection; for example, pneumonia, pyelonephritis, urinary tract infection, diverticulitis, meningitis, and cellulitis, among others. Two trained research assistants independently conducted a structured review of all retrieved medical records, including ED physician and nursing, admission, progress and discharge notes, laboratory reports, vital sign tables, and radiology reports. We reviewed records for the first 28 hours of hospitalization to allow for ED treatment plus one full day of hospitalization. Sepsis events were defined as a hospitalization for an infection with the presence of ≥2 systemic inflammatory response syndrome (SIRS) criteria (temperature <36 or >38 °C, heart rate >90 beats per minute, respiratory rate >20 breaths per minute or PaCO_2_ < 32 mmHg, white blood cell count <4000 or >12,000 cell/mm^3^) during the first 28 hours of hospitalization. We did not consider sepsis events occurring at later points of hospitalization. We assessed sepsis events occurring over a 10-year time period from January 1, 2003 through December 31, 2012.

### Participant and hospitalization characteristics

Participant characteristics were determined during the initial interview and in-home visit. Demographics included age, race, sex, income, education, and geographic location. Race was defined as White or Black/African American, and was self-reported. Income was divided into four categories (<$20,000, $20,000–34,000, $35,000–74,000, ≥$75,000). Education categories included less than high school, high school graduate, some college, and college or higher. Geographic region included the “stroke buckle” (coastal plains of North Carolina, South Carolina, and Georgia), the “stroke belt” (remainder of North Carolina, South Carolina, and Georgia plus Tennessee, Mississippi, Alabama, Louisiana, and Arkansas), and nonbelt. Health behaviors included cigarette smoking (current, past, never) and alcohol use (moderate use (1 drink per day for women or 2 drinks per day for men), heavy use (>1 drink per day for women and >2 drinks per day for men) as per the National Institute on Alcohol Abuse and Alcoholism classification).

Chronic medical conditions included cancer, CAD, diabetes, hypertension, obesity, and stroke. Participants self-reported history of cancer, myocardial infarction, or stroke. CAD included a history of myocardial infarction or electrocardiogram evidence of prior MI [19]. Diabetes was defined as fasting glucose ≥126 mg/L (or glucose ≥200 mg/L for those not fasting) or the use of insulin or oral hypoglycemic agents. Hypertension included systolic blood pressure ≥ 140 mmHg, diastolic blood pressure ≥ 90 mmHg, or the reported use of antihypertensive agents. Obesity included a body mass index ≥ 30 kg/m^2^ or waist circumference >102 cm for males or >88 cm for females.

REGARDS did not collect information on pulmonary conditions such as asthma and chronic obstructive pulmonary disease. Therefore, we defined participant use of pulmonary medications as a surrogate for chronic lung disease. Obtained from each participant’s medication inventory, pulmonary medications included beta-2 adrenergic agonists, leukotriene inhibitors, inhaled corticosteroids, combination inhalers, and other pulmonary medications such as ipratropium, cromolyn, aminophylline, and theophylline. Statin use was defined in a similar manner, with specific statins reported by subjects including atorvastatin, fluvastatin, lovastatin, pravastatin, rosuvastatin, and simvastatin. In order to account for medication adherence, we also obtained participant responses to the four-question version of the Morisky Medication Adherence Scale, which provides a measure of individual compliance with medication use (scale ranges from 0 to 4, with 0 indicating good adherence and 4 indicating poor adherence) [20].

We used data on biomarkers obtained for all subjects, including high-sensitivity C-reactive protein (hs-CRP), cystatin C, urinary albumin-to-creatinine ratio (ACR), and estimated glomerular filtration rate (eGFR). Study personnel collected blood and urine samples from all REGARDS participants at subjects’ homes following a 10–12-hour fast. Samples were centrifuged to separate serum or plasma within 2 hours of collection and shipped overnight on ice packs to the laboratories at the University of Vermont. On arrival, study personnel centrifuged the samples at 30,000 × *g* and 4 °C. The samples were either analyzed (general chemistries) immediately or stored at −80 °C.

Functional status measures included the 12-Item Short Form Health Survey (SF-12) physical composite score (PCS) in addition to self-reported physical activity and exhaustion. Consistent with established definitions, we defined physical weakness as PCS < 75, low weekly physical activity as responses of “almost never” or “never” to the question “How many times per week do you engage in intense physical activity, enough to work up a sweat?,” and exhaustion as responses of “a little of the time” or “none of the time” to the question “How much of the time during the past 4 weeks did you have energy?” [21–23].

Among participants hospitalized with sepsis, we identified several hospitalization characteristics via chart review of available clinical data. We identified sepsis events with elevated Sequential (sepsis-related) Organ Failure Assessment (SOFA) score (≥2 points) using established criteria [24, 25]. We also identified admission destination and in-hospital mortality.

### Primary exposure

Serum total cholesterol, HDL-C, and triglyceride levels were directly measured from serum samples. LDL-C was calculated using the Friedewald formula from total cholesterol, HDL-C, and triglyceride [26]. Our primary exposures were HDL-C and LDL-C quartiles. We also classified lipid levels using established clinical cutoff values for HDL-C (<40, 40–59, and ≥60 mg/dl) and LDL-C (<130, 130–159, and ≥160 mg/dl) [1]. We also analyzed lipid values as continuous variables and as percentages of total cholesterol (per standard deviation increase).

### Data analysis

We compared baseline participant characteristics across HDL-C and LDL-C quartiles using Pearson chi-square tests of association and *t* tests of equal means for categorical and continuous variables, respectively. Because we observed visual trends in biomarker levels based on lipid level quartiles, we specified tests for linear trend using modified Poisson regression for a common outcome, including abnormal biomarker as the dependent variable with HDL-C or LDL-C quartile as the independent variable.

In order to determine the association between LDL-C and HDL-C and rates of sepsis, we fit a series of Cox proportional hazards regression models, fitting time to first sepsis event as the outcome and lipid quartile as the exposure. We defined person-time at risk as days from first interview to first sepsis hospitalization. Participants without a sepsis event were censored at the earliest of last follow-up interview, death, or December 31, 2012. We sequentially adjusted the model for subject demographics (age, sex, race, education, income, geographic region), health behaviors (smoking or alcohol use), chronic medical conditions (cancer, chronic lung disease, CAD, diabetes, hypertension, obesity, stroke, statin use, Morisky adherence index), biomarkers (hs-CRP, cystatin C, ACR, eGFR), and functional status (weakness, exhaustion, low physical activity). We additionally examined HDL-C and LDL-C using relevant clinical cutoff values and as standardized continuous variables, both in their original forms and as a percentage of total cholesterol. We also stratified the analysis by statin use, generating new quartile cutoff values relative to nonusers and users. Lastly, we performed a sensitivity analysis in which several measures (body mass index, hs-CRP, cystatin C, ACR, and eGFR) were included as continuous variables.

Because of the substantial number of missing values for select variables included in our final model (HDL-C 4.8%, LDL-C 6.1%, cystatin C 6.8%, hs-CRP 6.4%, ACR 4.7%, eGFR 4.3%), we performed all modeling using multiple imputation. We implemented multiple imputation using chained equations with the Stata ‘MI’ suite, generating 10 imputed data sets and pooling estimates using Rubin’s rules [27, 28]. Patterns of missingness were examined in order to ensure that the assumption of randomness was met and the imputation model included all variables from the final analytic model, in addition to the Nelson–Aalen cumulative hazard function (a common approach for incorporating the outcome measure) [29].We ultimately reported adjusted hazard ratios and Kaplan–Meier failure functions after pooling across imputations. All analyses were performed using Stata 13.1 (Statacorp, College Station, TX, USA).

## Results

Of the 30,239 total REGARDS participants, we included 29,690 with complete follow-up information. Among this group, there were 3423 hospitalizations for serious infections, with 1845 total sepsis events among 1526 individuals. Median follow-up time was 3.6 years (IQR 1.8–5.6) for participants with first sepsis events and 6.6 years (IQR 5.2–8.1) for censored participants.

Baseline participant characteristics by lipid level quartile are presented in Table 1. Compared with the highest quartile, participants in the lowest HDL-C quartile were more likely to be male, White, have less than a high school education, reside in the stroke belt, be a current smoker, and use no alcohol. This group was also more likely to have a number of chronic medical conditions and biomarker abnormalities, including CAD, diabetes, hypertension, obesity, stroke, statin use, elevated hs-CRP/cystatin-C/ACR, and reduced eGFR. Similarly, compared with the highest quartile, participants in the lowest quartile for LDL-C were more likely to be male and White; also experiencing disproportionate CAD, diabetes, hypertension, stroke, statin use, elevated cystatin C/ACR, and reduced eGFR. In contrast to HDL-C, participants in the lowest LDL-C quartile were less likely to have elevated hs-CRP compared with the highest quartile. Significant linear trends across quartiles were observed for all biomarkers examined (*p* < 0.001).

**Table 1:** Baseline participant characteristics by HDL-C and LDL-C quartile

Characteristic	HDL-C quartile				LDL-C quartile			
	Q1, 5–40 mg/dl	Q2, 41–49 mg/dl	Q3, 50–61 mg/dl	Q4, 62–199 mg/dl	Q1, 3–89 mg/dl	Q2, 90–111 mg/dl	Q3, 112–135 mg/dl	Q4, 136–388 mg/dl
Number of participants	7305	7064	7162	6749	6984	7088	6915	6896
Demographics								
Age (years), mean (SD)	64.8 (9.3)	64.8 (9.5)	64.8 (9.3)	65.1 (9.5)	66.3 (9.5)	65.4 (9.5)	64.5 (9.4)	63.4 (9.0)
Age group (%)								
45–49	5.0	5.5	4.8	4.6	4.4	4.7	5.4	5.4
50–59	26.0	25.5	26.5	26.8	20.8	24.7	27.9	31.3
60–69	38.2	38.3	37.8	36.3	37.0	37.7	37.1	38.5
70–79	24.3	23.4	24.2	24.6	28.9	25.1	23.0	19.8
≥80	6.6	7.3	6.6	7.7	8.8	7.9	6.7	5.0
Gender (%)								
Male	71.3	51.6	35.3	21.7	51.1	47.1	43.6	38.9
Female	28.7	48.4	64.7	78.3	48.9	52.9	56.4	61.1
Race (%)								
White	67.6	58.8	55.7	55.5	62.3	61.3	58.6	54.6
Black	32.4	41.3	44.3	44.5	37.7	38.7	41.4	45.5
Education (%)								
Less than high school	13.1	12.8	12.4	10.9	13.5	11.8	11.8	12.1
High school graduate	25.8	26.4	26.4	24.6	26.1	25.4	24.4	27.3
Some college	27.2	26.6	26.9	26.5	26.0	26.7	27.8	26.7
College or higher	34.0	34.2	34.3	38.1	34.5	36.2	36.1	33.9
Missing (*N*)	7	4	3	7	7	4	3	7
Income (%)								
< $20,000	16.6	18.3	18.0	17.7	18.2	16.9	16.5	18.7
$20,000–34,000	24.0	24.0	24.9	23.5	25.2	24.0	22.9	24.4
$35,000–74,000	32.1	30.8	29.1	28.0	29.8	30.8	30.7	29.1
≥ $75,000	16.6	15.5	15.5	16.4	15.0	16.2	17.3	15.6
Refused	10.7	11.4	12.5	14.4	11.9	12.1	12.6	12.2
Geographic region (%)								
Stroke buckle	20.7	20.6	21.7	20.9	22.4	20.7	20.4	20.2
Stroke belt	37.0	35.7	33.7	32.6	35.1	35.1	33.7	34.9
Nonbelt/buckle	42.3	43.7	44.7	46.5	42.5	44.2	46.0	44.9
Health behaviors								
Smoking status (%)								
Current	17.1	15.1	13.2	12.1	14.1	13.1	14.2	15.8
Past	44.9	41.1	38.3	37.1	44.4	40.9	39.1	36.9
Never	38.1	43.9	48.5	50.9	41.5	46.0	46.7	47.2
Missing (*N*)	17	29	31	28	23	24	31	25
Alcohol use (%)								
Heavy	1.8	3.1	4.0	7.5	4.0	4.4	3.9	3.7
Moderate	31.8	32.5	33.5	36.7	32.3	35.0	34.8	32.0
None	66.4	64.4	62.5	55.9	63.7	60.6	61.3	64.3
Missing (*N*)	155	153	106	134	145	140	122	130
Chronic medical conditions								
Cancer (%)	10.3	8.5	8.0	8.0	10.2	9.3	8.0	7.2
Chronic lung disease (%)	8.3	9.2	9.2	10.0	10.7	9.7	8.8	7.4
Coronary artery disease (%)	25.3	19.2	15.1	11.5	28.7	18.7	12.9	10.7
Diabetes (%)	30.3	25.4	19.0	14.2	33.5	22.9	17.0	14.8
Hypertension (%)	63.5	60.8	58.0	53.7	67.0	60.2	55.9	52.3
Obesity (%)	61.7	57.2	53.6	40.0	54.5	52.0	52.3	54.0
Stroke (%)	7.6	7.0	5.7	4.4	8.5	6.3	5.0	4.9
Statin use (%)	35.3	35.2	31.6	23.6	56.6	47.0	36.8	37.0
Biomarkers								
hs-CRP > 3.0 mg/dl	44.6	41.8	40.3	35.5	38.3	39.6	40.8	43.8
Missing (*N*)	148	165	167	179	150	177	159	160
Cystatin C > 1.12 mg/dl	34.1	26.7	21.6	17.3	32.1	25.7	22.2	19.7
Missing (*N*)	183	187	208	212	189	199	196	191
ACR > 30 μg/mg (%)	18.8	15.7	13.0	12.5	17.5	15.0	13.7	13.3
Missing (*N*)	267	308	249	254	295	287	227	250
eGFR < 60 ml/min/1.73 m^2^	14.7	11.8	10.3	8.7	15.2	11.9	9.7	8.8
Missing (*N*)	1	0	1	0	0	0	1	1
Functional status								
Weakness (SF-12 PCS < 75) (%)	31.0	29.9	28.8	26.7	33.8	28.9	26.5	26.8
Reported exhaustion (%)	15.7	13.9	13.4	11.7	14.8	14.1	12.4	13.0
Low physical activity (%)	34.7	33.5	33.6	32.6	35.4	33.7	32.6	32.6

Total of 29,690 participants. LDL-C measurement missing for 1807 participants. HDL-C measurement missing for 1410 participants

All *p* < 0.05 based on Pearson chi-square tests of association

*LDL-C* low-density lipoprotein, *HDL-C* high-density lipoprotein, *SD* standard deviation, *hs-CRP* high-sensitivity C-reactive protein, *ACR* albumin–creatinine ratio, *eGFR* estimated glomerular filtration rate, *SF-12* 12-item short form survey, *PCS* physical composite score

Sepsis incidence differed substantially across HDL-C and LDL-C quartiles, with the highest incidence observed in the lowest quartile and the lowest incidence in the highest quartile for both lipid types (Table 2). The most common infection types associated with first sepsis episode were pneumonia (39.4%), kidney and urinary tract (17.0%), and abdominal infections (15.1%). Pneumonia was more common for participants in the highest quartile of HDL-C compared with the lowest, while abdominal infections were more common in the lowest quartile. For LDL-C, kidney and urinary tract infections were more common in the lowest quartile compared with the highest. Compared with the higher quartiles, the lowest quartiles of HDL-C and LDL-C had higher proportions of individuals with elevated SOFA scores.

**Table 2:** Sepsis incidence rates and hospitalization characteristics by HDL-C and LDL-C quartile

Variable	HDL-C quartile				*p*	LDL-C quartile				*p*
	Q1, 5–40 mg/dl	Q2, 41–49 mg/dl	Q3, 50–61 mg/dl	Q4, 62–199 mg/dl		Q1, 3–89 mg/dl	Q2, 90–111 mg/dl	Q3, 112–135 mg/dl	Q4, 136–388 mg/dl	
Number of participants	7305	7064	7162	6749		6984	7088	6915	6896	
Sepsis events, *N* (%)	485 (6.6)	372 (5.3)	329 (4.6)	264 (3.9)		451 (6.5)	399 (5.6)	304 (4.4)	261 (3.8)	
IR (per 1000 person-years), 95% CI	10.6 (9.7–11.6)	8.5 (7.7–9.4)	7.3 (6.6–8.1)	6.2 (5.5–7.0)		10.6 (9.6–11.6)	8.9 (8.1–9.9)	6.9 (6.2–7.7)	6.0 (5.3–6.8)	
Sepsis event hospitalization characteristics										
Infection type, *N* (%)										
Pneumonia	179 (36.9)	128 (34.4)	138 (42.0)	120 (45.5)	0.018	181 (40.1)	151 (37.8)	113 (37.2)	105 (40.2)	0.79
Kidney and urinary tract infections	86 (17.7)	71 (19.1)	58 (17.6)	22 (12.5)	0.16	80 (17.7)	79 (19.8)	61 (20.1)	22 (8.4)	<0.001
Abdominal	78 (16.1)	71 (19.1)	40 (12.2)	22 (12.5)	0.038	62 (13.8)	64 (16.0)	44 (14.5)	47 (18.0)	0.45
Skin and soft tissue	50 (10.3)	29 (7.8)	17 (5.2)	20 (7.6)	0.066	43 (9.5)	26 (6.5)	26 (8.6)	19 (7.3)	0.40
Bronchitis, influenza and other lung infections	34 (7.0)	34 (9.1)	35 (10.6)	31 (11.7)	0.13	34 (7.5)	38 (9.5)	34 (11.2)	27 (10.3)	0.35
Sepsis	42 (8.7)	21 (5.7)	18 (5.5)	16 (6.1)	0.20	30 (6.7)	23 (5.8)	16 (5.3)	24 (9.2)	0.24
Fever of unknown origin	4 (0.8)	7 (1.9)	11 (3.3)	4 (1.5)	0.072	6 (1.3)	9 (2.3)	3 (1.0)	7 (2.7)	0.34
Surgical wound	1 (0.2)	3 (0.8)	5 (1.5)	1 (0.4)	0.14	2 (0.4)	2 (0.5)	2 (0.7)	4 (1.5)	0.41
Catheter (IV/central/dialysis)	2 (0.4)	0 (0)	0 (0)	3 (1.1)	–	2 (0.4)	1 (0.3)	1 (0.3)	0 (0)	0.91
Meningitis	0 (0)	2 (0.5)	2 (0.6)	1 (0.4)	0.27	2 (0.4)	1 (0.3)	0 (0)	2 (0.8)	0.51
Unknown/other	9 (1.9)	6 (1.6)	5 (1.5)	2 (0.8)	0.70	9 (2.0)	5 (1.3)	4 (1.3)	4 (1.5)	0.85
Elevated SOFA score (SOFA ≥ 2), *N* (%)	266 (54.9)	175 (47.0)	147 (44.7)	119 (45.1)	0.010	251 (55.7)	201 (50.4)	127 (41.8)	107 (41.0)	<0.001
Admitted to ICU vs floor, *N* (%)^a^	69 (16.0)	42 (12.8)	41 (14.4)	24 (9.9)	0.16	70 (17.0)	41 (11.6)	26 (10.2)	32 (13.9)	0.051
In-hospital death, *N* (%)	43 (8.9)	30 (8.1)	30 (9.1)	25 (9.5)	0.93	43 (9.5)	35 (8.8)	19 (6.3)	29 (11.1)	0.22

Total of 29,690 participants. LDL-C measurement missing for 1807 participants. HDL-C measurement missing for 1410 participants

*p* values from Pearson chi-square tests of association for variables with at least five observations in 75% of table cells, and Fisher exact tests for all others

^a^Includes only events resulting in an inpatient admission and those with inpatient records available

*LDL-C* low-density lipoprotein, *HDL-C* high-density lipoprotein, *IR* incidence rate, *CI* confidence interval, *SOFA* Sepsis-related Organ Failure Assessment, *ICU* intensive care unit

**Fig. 1:**
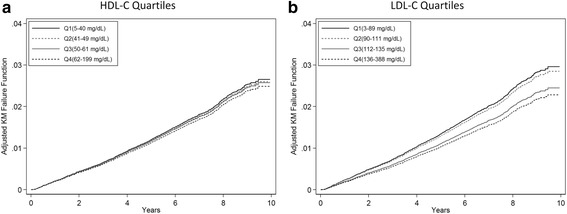
Adjusted Kaplan–Meier (*KM*) failure curves for time to sepsis by HDL-C and LDL-C quartiles (Q1 = lowest quartile, Q4 = highest quartile). Total of 29,690 participants. (**a**) High-density lipoprotein cholesterol (*HDL-C*) and (**b**) low-density lipoprotein cholesterol (*LDL-C*). All failure functions estimated among White, female, nonsmoking and non-alcohol-consuming participants residing in the nonbelt region, with no history of comorbidities, normal biomarker levels, and no functional status impairments (all binary variables set to zero and all categorical variables set to the reference groups)

**Fig. 2:**
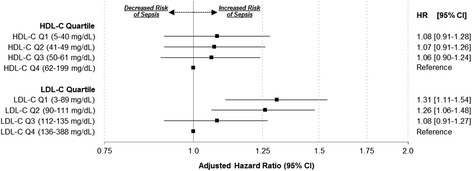
Risk of sepsis by HDL-C or LDL-C quartile (Q1 = lowest quartile, Q4 = highest quartile). *LDL-C* low-density lipoprotein, *HDL-C* high-density lipoprotein, *HR* hazard ratio, *CI* confidence interval

In unadjusted analyses, the lowest quartiles for both HDL-C and LDL-C were associated with increased sepsis rates (Table 3 and Fig. 1). After adjustment for demographics, health behaviors, and chronic medical conditions, HDL-C quartile remained associated with long-term risk of sepsis. However, after the addition of biomarkers to the model, HDL-C was no longer associated with rates of sepsis. Similarly, HDL-C defined using clinical cutoff values or as a continuous variable showed no associations with sepsis risk. However, LDL-C in the lowest two quartiles was associated with increased adjusted rates of sepsis. We observed a linear trend across LDL-C quartiles (Figs. 1 and 2). We obtained similar inferences when LDL-C was defined as a continuous variable or as a percentage of total cholesterol. We observed similar results when stratifying by statin use and redefining quartile cutoff values (Table 4). Lastly, we observed similar associations when adjusting for BMI, hs-CRP, Cystatin C, ACR, and eGFR as continuous variables. (Additional file 1: Table S1)

**Table 3:** Associations of HDL-C and LDL-C with sepsis rates

					Include HDL-C + LDL-C in same model			
Variable	Unadjusted	Add demographics + health behaviors + chronic medical conditions	Add biomarkers	Add functional status measures	HDL-C and LDL-C quartiles	HDL-C and LDL-C clinical categories	HDL-C and LDL-C SD	HDL-C and LDL-C percent of total cholesterol
HDL-C quartile								
Q1 (5–40 mg/dl)	1.71 (1.48–1.98)	1.22 (1.03–1.44)	1.08 (0.91–1.27)	1.07 (0.90–1.26)	1.08 (0.91–1.28)			
Q2 (41–49 mg/dl)	1.37 (1.17–1.60)	1.11 (0.94–1.32)	1.05 (0.89–1.24)	1.05 (0.89–1.23)	1.07 (0.91–1.26)			
Q3 (50–61 mg/dl)	1.18 (1.01–1.39)	1.07 (0.91–1.25)	1.04 (0.88–1.22)	1.04 (0.88–1.22)	1.06 (0.90–1.24)			
Q4 (62–199 mg/dl)	Reference	Reference	Reference	Reference	Reference			
Trend *p* value	<0.001	0.02	0.40	0.46	0.39			
HDL-C clinical category								
<40 mg/dl	1.69 (1.47–1.95)	1.20 (1.02–1.41)	1.05 (0.89–1.24)	1.05 (0.89–1.23)		1.05 (0.89–1.24)		
40–59 mg/dl	1.25 (1.10–1.43)	1.07 (0.93–1.23)	1.01 (0.88–1.16)	1.01 (0.88–1.16)		1.03 (0.82–1.29)		
≥60 mg/dl	Reference	Reference	Reference	Reference		Reference		
HDL-C (per SD) (mg/dl)	0.83 (0.79–0.88)	0.96 (0.90–1.02)	1.01 (0.95–1.08)	1.01 (0.95–1.08)			1.01 (0.95–1.07)	
HDL-C % of total cholesterol (per SD)	0.95 (0.91–1.01)	1.01 (0.96–1.07)	1.06 (1.01–1.13)	1.06 (1.01–1.13)				1.01 (0.94–1.09)
LDL-C quartile								
Q1 (3–89 mg/dl)	1.70 (1.47–1.97)	1.29 (1.10–1.52)	1.31 (1.12–1.54)	1.30 (1.11–1.53)	1.31 (1.11–1.54)			
Q2 (90–111 mg/dl)	1.42 (1.21–1.66)	1.25 (1.06–1.47)	1.26 (1.07–1.48)	1.25 (1.06–1.48)	1.26 (1.06–1.48)			
Q3 (112–135 mg/dl)	1.13 (0.97–1.33)	1.07 (0.91–1.25)	1.08 (0.91–1.26)	1.07 (0.91–1.26)	1.08 (0.91–1.27)			
Q4 (136–388 mg/dl)	Reference	Reference	Reference	Reference	Reference			
Trend *p* value	<0.001	0.001	<0.001	<0.001	<0.001			
LDL-C clinical category								
<130 mg/dl	1.49 (1.23–1.81)	1.23 (1.01–1.51)	1.27 (1.04–1.55)	1.26 (1.03–1.54)		1.26 (1.03–1.55)		
130–159 mg/dl	1.02 (0.82–1.28)	1.00 (0.80–1.26)	1.03 (0.82–1.29)	1.03 (0.82–1.29)		1.03 (0.82–1.29)		
≥160 mg/dl	Reference	Reference	Reference	Reference		Reference		
LDL-C (per SD) (mg/dl)	0.81 (0.76–0.85)	0.90 (0.85–0.96)	0.90 (0.85–0.95)	0.90 (0.85–0.96)			0.90 (0.85–0.96)	
LDL-C % of total cholesterol (per SD)	0.86 (0.81–0.90)	0.92 (0.87–0.97)	0.92 (0.87–0.97)	0.92 (0.87–0.97)				0.93 (0.86–0.99)

Data presented as hazard ratio (95% confidence interval). Total of 29,690 REGARDS participants. Hazard ratios estimated using Cox proportional hazards regression and represent pooled estimates from multiple imputation

*p* values represent tests for linear trends across quartiles

Demographics = age, gender, race, region, income, and education; health behaviors = smoking status and alcohol use; chronic medical conditions = cancer, chronic lung disease, coronary artery disease, diabetes, hypertension, obesity, stroke, statin use, and Morisky medication adherence index; biomarkers = hs-CRP, Cystatin C, ACR, and eGFR; functional status measures = weakness, exhaustion, and low physical activity

*LDL-C* low-density lipoprotein, *HDL-C* high-density lipoprotein, *SD* standard deviation, *hs-CRP* high-sensitivity C-reactive protein, *ACR* albumin–creatinine ratio, *eGFR* estimated glomerular filtration rate

**Table 4:** Associations of HDL-C and LDL-C with sepsis rates stratified by statin use

Variable	Total *N*	Sepsis events,	Sepsis incidence (per 1000 person-years),	Models for sepsis hazard	
				Unadjusted,	Adjusted^a^,
		*N* (%)	IR (95% CI)	HR (95% CI)	HR (95% CI)
Statin non-users					
HDL-C quartile					
Q1 (5–41 mg/dl)	5233	315 (6.0)	9.6 (8.6–10.7)	1.73 (1.43–2.09)	1.09 (0.88–1.36)
Q2 (42–50 mg/dl)	4540	223 (4.9)	7.9 (6.9–9.0)	1.43 (1.16–1.76)	1.11 (0.89–1.37)
Q3 (51–62 mg/dl)	4751	201 (4.2)	6.8 (5.9–7.8)	1.21 (0.98–1.49)	1.06 (0.86–1.31)
Q4 (63–199 mg/dl)	4837	169 (3.5)	5.5 (4.8–6.4)	Reference	Reference
			Trend *p* value	<0.001	0.44
LDL-C quartile					
Q1 (3–99 mg/dl)	4816	278 (5.8)	9.4 (8.3–10.5)	1.54 (1.28–1.86)	1.32 (1.10–1.60)
Q2 (100–120 mg/dl)	4810	231 (4.8)	7.6 (6.7–8.7)	1.29 (1.06–1.57)	1.19 (0.98–1.46)
Q3 (121–143 mg/dl)	4842	212 (4.4)	6.9 (6.0–7.9)	1.13 (0.93–1.37)	1.11 (0.91–1.36)
Q4 (144–388 mg/dl)	4619	169 (3.7)	5.8 (5.0–6.8)	Reference	Reference
			Trend *p* value	<0.001	0.003
Statin users					
HDL-C quartile					
Q1 (11–39 mg/dl)	2304	170 (7.4)	11.8 (10.2–13.7)	1.37 (1.08–1.73)	0.89 (0.69–1.17)
Q2 (40–47 mg/dl)	2274	134 (5.9)	9.6 (8.1–11.4)	1.11 (0.87–1.43)	0.87 (0.67–1.13)
Q3 (48–57 mg/dl)	2163	119 (5.5)	8.8 (7.3–10.5)	1.03 (0.80–1.33)	0.93 (0.72–1.20)
Q4 (58–156 mg/dl)	2178	119 (5.5)	8.7 (7.3–10.4)	Reference	Reference
			Trend *p* value	0.006	0.39
LDL-C quartile					
Q1 (11–76 mg/dl)	2210	175 (7.9)	13.1 (11.3–15.1)	2.00 (1.55–2.59)	1.50 (1.14–1.96)
Q2 (77–93 mg/dl)	2304	142 (6.2)	9.9 (8.4–11.7)	1.55 (1.19–2.03)	1.36 (1.03–1.78)
Q3 (94–111 mg/dl)	2091	127 (6.1)	9.6 (8.1–11.4)	1.49 (1.13–1.96)	1.38 (1.04–1.81)
Q4 (112–315 mg/dl)	2191	81 (3.7)	5.9 (4.7–7.3)	Reference	Reference
			Trend *p* value	<0.001	0.008

Total of 29,690 REGARDS participants. HRs estimated using Cox proportional hazards regression and represent pooled estimates from multiple imputation

*p* values represent tests for linear trends across quartiles

^a^Adjusted for demographics, health behaviors, chronic medical conditions, biomarkers, and functional status measures. Demographics = age, gender, race, region, income, and education; health behaviors = smoking status and alcohol use; chronic medical conditions = cancer, chronic lung disease, coronary artery disease, diabetes, hypertension, obesity, stroke, and Morisky medication adherence index; biomarkers = hs-CRP, Cystatin C, ACR, and eGFR; functional status measures = weakness, exhaustion, and low physical activity

*HR* hazard ratio, *IR* incidence rate, *CI* confidence interval, *LDL-C* low-density lipoprotein, *HDL-C* high-density lipoprotein, *hs-CRP* high-sensitivity C-reactive protein, *ACR* albumin–creatinine ratio, *eGFR* estimated glomerular filtration rate

## Discussion

In this large, prospective cohort study we have demonstrated that baseline cholesterol levels may be associated with long-term risk of sepsis. Specifically, we found that lower LDL-C values, though generally accepted to be favorable in terms of cardiovascular disease risk and prevention of CAD, seem to be associated with increased long-term risk of sepsis. Contrary to our original hypothesis, we found no association between low HDL-C and sepsis risk after adjusting for potential confounders.

In recent years, the bulk of sepsis research has focused on early detection and care of acute sepsis. But few efforts have conceptualized sepsis as a preventable condition. Identifying risk factors and developing a strategy for sepsis prevention could be a worthwhile endeavor to reduce the societal burden of this life-threatening and costly disease. The results of this study have important, yet perplexing implications for sepsis prevention. In the cardiovascular literature, lower LDL-C (<130 mg/dl) and higher HDL-C (>60 mg/dl) thresholds are thought to be protective against CAD [1]. Our results, however, demonstrate that lower LDL-C levels are associated with increased long-term risk of sepsis. We have also demonstrated an association between low LDL-C and HDL-C and elevated SOFA scores.

Previous studies have demonstrated that low cholesterol levels are a risk factor for sepsis [16, 30, 31] and have also shown that lipid levels are rapidly changing during early sepsis and may predict outcomes [12–14, 30, 31]. In the setting of infection, cholesterol levels may drop dramatically because of decreases in LDL-C and HDL-C. The exact mechanism for this is unknown; however, bacterial endotoxin, tumor necrosis factor, interleukin-2, and interferon beta, all potentially present during systemic infection, reduce serum cholesterol levels [32–37]. Additionally, both LDL-C and HDL-C play a proven role in clearance of bacterial toxins, lipopolysaccharide (LPS) from Gram-negative bacteria, and lipoteichoic acid from Gram-positive bacteria. In studies where LPS was added to human whole blood in vitro, LPS was bound to HDL-C (60%), LDL-C (25%), and very low density lipoprotein (VLDL)-cholesterol (12%) [38]. Therefore, one potential explanation for the increased long-term sepsis risk with low LDL-C is the inability to clear bacterial toxins from the bloodstream.

Interestingly, our study did not demonstrate an association between low HDL-C and long-term sepsis risk. At a glance, these results appear contrary to findings by Chien et al. that low HDL-C levels in acute sepsis are predictive of increased 30-day mortality, prolonged ICU stay (> 7 days), and increased hospital-acquired infections. However, there are two reasons why these results should not be entirely surprising. First, recent evidence has demonstrated that HDL-C function may be more important than quantity. Examples include recent clinical trials that failed to show benefit to medically elevating HDL-C to prevent myocardial infarction [3]. Another recent Mendelian randomization study failed to find a reduced risk of myocardial infarction in a group of patients with a specific single nucleotide polymorphism predisposing to elevated HDL-C [39]. These studies are timely, given a burgeoning area of research demonstrating that HDL-C can become dysfunctional and proinflammatory under certain chronic (metabolic syndrome, diabetes mellitus, rheumatoid arthritis, CAD) and acute (sepsis, myocardial infarction, stroke) inflammatory conditions [40–44]. HDL-C function may then explain the lack of a protective effect in this study, given the prevalence of chronic inflammatory conditions within the REGARDS cohort. Another explanation may be the difference between rapid lowering of lipid levels during acute inflammatory states which relate to disease severity, rather than chronically low levels of HDL-C which, if dysfunctional, may not protect as well against sepsis. In order to address this question, future studies aimed at prospectively measuring dysfunctional HDL and monitoring for the development of sepsis are needed.

Potential clinical applications for the results of this study may include modulation of LDL clearance. Lipid-based pathogens from bacteria are first bound to binding proteins which are then bound by HDL and transferred to LDL or VLDL molecules. LDL and VLDL are then cleared from the bloodstream by hepatocytes via the LDL receptor. The proprotein convertase subtilisin/kexin type 9 (PCSK9) molecule binds the LDL receptor on hepatocytes, and promotes internalization and lysosomal degradation of the receptor, thus preventing clearance of LDL and VLDL from the blood [45]. PCSK9 inhibitors are currently under investigation as lipid-lowering agents because they promote LDL clearance and thus reduce LDL levels, but they may also have an application in clearance of lipid-based pathogens in sepsis. PCSK9 inhibition results in decreased inflammatory cytokine production and physiological responses to endotoxin in septic mice and increased PCSK9 levels are associated with reduced endotoxin clearance and organ failure in sepsis [45, 46]. Although this is a new area of research, it demonstrates that modulation of cholesterol metabolism may have potential as a future application of these findings. In addition, because HDL plays a critical intermediary step in lipid-based pathogen clearance, investigating HDL function in sepsis is of increased importance. If the association between HDL-C and sepsis-associated organ dysfunction is valid, studies of HDL-based therapies including apoliprotein mimetic peptides and synthetic HDL may hold potential [47, 48].

In our analysis, we chose to stratify by baseline statin use, because statins have had significant attention in the literature with regards to protection against sepsis. The mechanisms by which statins may protect against sepsis are unknown, though a new class of lipid mediators called resolvins may play a role [49]. Also, varying protection against sepsis has been found between different statins, with atorvastatin being the most promising [50]. Our group completed a recent study which did not find baseline statin use to be protective against long-term sepsis in the REGARDS cohort [51]. In addition, statin use did not significantly influence our results with regards to baseline lipid levels. Two recent meta-analyses of only randomized, controlled trials also found no benefit to statin use in sepsis [52, 53].

This study had several limitations. Only baseline lipid levels were available for analysis, there were no repeat levels or levels prior to the sepsis event. Therefore, lipid levels may have changed over time prior to the sepsis event, though we feel that the magnitude of change would have been unlikely to change our results. REGARDS was not designed to study sepsis, and therefore some sepsis events could have been missed. However, given the systematic hospital record review, we believe we have minimized the potential for missed or wrongly identified cases of sepsis. The REGARDS cohort only includes Black and White participants at least 45 years of age, and therefore results may not be as generalizable to other ethnic groups or a younger population. Sepsis events were limited to community-acquired sepsis and did not include hospital-acquired sepsis events, because hospital sepsis is more likely to be influenced by multiple factors related to the hospitalization. Also, while we made every effort to adjust for potential confounders, other variables such as vaccination or access to healthcare which were not accounted for may have influenced the results. Finally, residual confounding is a potential concern, as with any observational study. We accounted for a comprehensive range of variables through our risk adjustment strategy for each participant at the beginning of the REGARDS study, but we could not account for changes in these patterns over time.

## Conclusions

In this large, prospective cohort study, low LDL-C was associated with increased long-term rates of community-acquired sepsis. HDL-C was not associated with long-term sepsis. Future studies should attempt to characterize the pathophysiologic or mechanistic basis for these associations.

## Additional file

Additional file 1: Table S1. Is presenting results analyzed using a nonimputed data set. *Adjusted for demographics, health behaviors, chronic medical conditions, biomarker values, and functional status measures. Demographics = age, gender, race, region, income, and education; health behaviors = smoking status and alcohol use; chronic medical conditions = cancer, chronic lung disease, coronary artery disease, diabetes, hypertension, obesity, stroke, and Morisky medication adherence index; biomarkers = hs-CRP, Cystatin C, ACR, and eGFR; functional status measures = weakness, exhaustion, and low physical activity. *HR* hazard ratio, *CI* confidence interval, *LDL-C* low-density lipoprotein cholesterol, *HDL-C* high-density lipoprotein cholesterol, *hs-CRP* high-sensitivity C-reactive protein, *ACR* albumin–creatinine ratio, *eGFR* estimated glomerular filtration rate. (DOCX 12 kb)

## Abbreviations

ACR: Urinary albumin-to-creatinine ratio
CAD: Coronary artery disease
ED: Emergency department
eGFR: Estimated glomerular filtration rate
HDL-C: High-density lipoprotein cholesterol
hs-CRP: High-sensitivity C-reactive protein
IQR: Interquartile range
LDL-C: Low-density lipoprotein cholesterol
PCS: Physical composite score
PCSK9: Proprotein convertase subtilisin/kexin type 9 molecule
REGARDS: Reasons for Geographic and Racial Disparities in Stroke
SF-12: 12-Item Short Form Health Survey
SIRS: Systemic inflammatory response syndrome criteria
SOFA: Sequential (sepsis-related) Organ Failure Assessment
VLDL: very low density lipoprotein
