# Supplementary material for: Cholesterol levels and long-term rates of community-acquired sepsis
Source: Crit Care. 2016 Dec 23;20:408. doi: 10.1186/s13054-016-1579-8 (PMC5180408; doi:10.1186/s13054-016-1579-8)
Supplement: Additional file 1: Table S1. — Is presenting results analyzed using a nonimputed data set. *Adjusted for demographics, health behaviors, chronic medical conditions, biomarker values, and functional status measures. Demographics = age, gender, race, region, income, and education; health behaviors = smoking status and alcohol use; chronic medical conditions = cancer, chronic lung disease, coronary artery disease, diabetes, hypertension, obesity, stroke, and Morisky medication adherence index; biomarkers = hs-CRP, Cystatin C, ACR, and eGFR; functional status measures = weakness, exhaustion, and low physical activity. HR hazard ratio, CI confidence interval, LDL-C low-density lipoprotein cholesterol, HDL-C high-density lipoprotein cholesterol, hs-CRP high-sensitivity C-reactive protein, ACR albumin–creatinine ratio, eGFR estimated glomerular filtration rate. (DOCX 12 kb) [file 13054_2016_1579_MOESM1_ESM.docx]

# Additional file 1

# TABLE S1

Associations of HDL-C and LDL-C with sepsis rates adjusted for continuous measures.

| **Variable** | **Unadjusted** | **Adjusted***  **(Dichotomous Variables)** | **Adjusted***  **(Continuous Variables)** | **Adjusted***  **HDL-C + LDL-C in Same Model**  **(Continuous Variables)** |
| --- | --- | --- | --- | --- |
|  | **HR (95% CI)** | **HR (95% CI)** | **HR (95% CI)** | **HR (95% CI)** |
| **HDL-C Quartile** |  |  |  |  |
| Q1 (5-40 mg/dL) | 1.71 (1.47-1.99) | 1.10 (0.91-1.33) | 1.17 (0.97-1.42) | 1.17 (0.96-1.42) |
| Q2 (41-49 mg/dL) | 1.36 (1.16-1.60) | 1.09 (0.91-1.31) | 1.13 (0.94-1.36) | 1.15 (0.96-1.39) |
| Q3 (50-61 mg/dL) | 1.17 (1.00-1.38) | 1.06 (0.89-1.28) | 1.07 (0.89-1.29) | 1.08 (0.90-1.31) |
| Q4 (62-199 mg/dL) | Ref | Ref | Ref | Ref |
|  |  |  |  |  |
| **LDL-C Quartile** |  |  |  |  |
| Q1 (3-89 mg/dL) | 1.76 (1.51-2.05) | 1.25 (1.04-1.51) | 1.22 (1.01-1.47) | 1.23 (1.02-1.48) |
| Q2 (90-111 mg/dL) | 1.49 (1.27-1.74) | 1.30 (1.09-1.55) | 1.29 (1.08-1.54) | 1.30 (1.09-1.55) |
| Q3 (112-135 mg/dL) | 1.15 (0.97-1.35) | 1.08 (0.90-1.29) | 1.07 (0.89-1.29) | 1.07 (0.89-1.29) |
| Q4 (136-388 mg/dL) | Ref | Ref | Ref | Ref |
|  |  |  |  |  |
